# Research on a Feedthrough Suppression Scheme for MEMS Gyroscopes Based on Mixed-Frequency Excitation Signals

**DOI:** 10.3390/mi16101120

**Published:** 2025-09-30

**Authors:** Xuhui Chen, Zhenzhen Pei, Chenchao Zhu, Jiaye Hu, Hongjie Lei, Yidian Wang, Hongsheng Li

**Affiliations:** 1 Xi’an Flight Automatic Control Research Institute of AVIC, Xi’an 710076, China; 15829700827@163.com; 2 School of Instrument Science and Engineering, Southeast University, Nanjing 210096, China; zhenpei244@163.com (Z.P.); zhuchenchao000925@163.com (C.Z.); hujiaye5@163.com (J.H.); hsli@seu.edu.cn (H.L.); 3 Key Laboratory of Micro-Inertial Instruments and Advanced Navigation Technology, Ministry of Education, Nanjing 210096, China; 4 State Key Laboratory of Comprehensive PNT Network and Equipment Technology, Nanjing 210096, China; 5 Aviation Industry Corporation of China, Xi’an 710076, China; hjlei@facri.com

**Keywords:** MEMS gyroscope, feedthrough interference, mixed-frequency excitation signals

## Abstract

Feedthrough interference is inevitably introduced in MEMS gyroscopes due to non-ideal factors such as circuit layout design and fabrication processes, exerting non-negligible impacts on gyroscope performance. This study proposes a feedthrough suppression scheme for MEMS gyroscopes based on mixed-frequency excitation signals. Leveraging the quadratic relationship between excitation voltage and electrostatic force in capacitive resonators, the resonator is excited with a modulated signal at a non-resonant frequency while sensing vibration signals at the resonant frequency. This approach achieves linear excitation without requiring backend demodulation circuits, effectively separating desired signals from feedthrough interference in the frequency domain. A mixed-frequency excitation-based measurement and control system for MEMS gyroscopes is constructed. The influence of mismatch phenomena under non-ideal conditions on the control system is analyzed with corresponding solutions provided. Simulations and experiments validate the scheme’s effectiveness, demonstrating feedthrough suppression through both amplitude-frequency characteristics and scale factor perspectives. Test results confirm the scheme eliminates the zero introduced by feedthrough interference in the gyroscope’s amplitude-frequency response curve and reduces force-to-rebalanced detection scale factor fluctuations caused by frequency split variations by a factor of 21. Under this scheme, the gyroscope achieves zero-bias stability of 0.3118 °/h and angle random walk of 0.2443 °/h/√Hz.

## 1. Introduction

Micro-Electro-Mechanical Systems (MEMS) gyroscopes have emerged as a cornerstone technology for inertial sensing across a broad spectrum of applications, from consumer electronics to high-precision navigation and autonomous platforms, where stringent requirements on bias stability, scale-factor linearity, and long-term robustness must be satisfied [1,2,3]. With performance specifications becoming increasingly stringent, parasitic coupling between drive and sense electrodes—commonly referred to as feedthrough—has become a major factor limiting attainable sensing accuracy and closed-loop control fidelity. Such feedthrough predominantly originates from fabrication- and layout-induced parasitics that give rise to capacitive coupling, which in turn introduces spurious components at the drive frequency into the sensing path, thereby distorting the frequency response and undermining scale-factor stability [4,5,6,7].

Several approaches have been investigated to mitigate feedthrough interference in MEMS gyroscopes. Structural and electrode optimization seeks to suppress mutual capacitance or geometrically cancel parasitic coupling through symmetric layouts, shielding structures, or modified electrode topologies. Representative implementations include differential comb geometries, grounded shielding layers, and electrode relocation to minimize direct capacitive paths [8,9]. While such methods can alleviate feedthrough during the design stage, they generally require substantial redesign effort and cannot fully eliminate residual coupling introduced by fabrication tolerances or environmental variations after deployment [10,11,12].

## 2. MEMS Gyroscope Modeling

### 2.1. Basic Architecture of MEMS Gyroscopes

**Figure 1:**
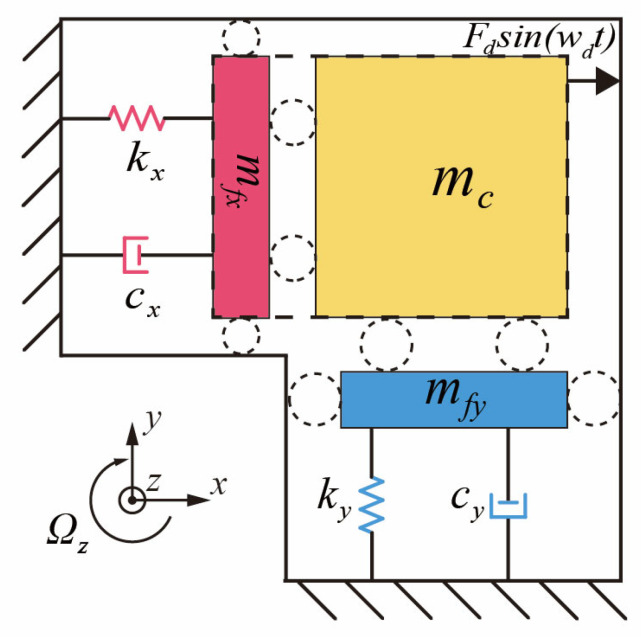
The typical MEMS vibratory gyroscope model.

As depicted in Figure 1, a basic MEMS vibratory gyroscope operates with uncoupled drive and sense modes, which can be modeled as a two-dimensional oscillator [13]. According to Newton’s second law, simplified dynamics equations for the drive mode and sense mode of the MEMS gyroscope are obtained as follows [14]:

(1) $$\begin{array}{l} m_{x}\ddot{x}+c_{x}\dot{x}+k_{x}x=F_{d}\sin(\omega_{d}t)\\ m_{y}\ddot{y}+c_{y}\dot{y}+k_{y}y=F_{y}-2m_{c}\Omega_{z}\dot{x}, \end{array}$$

where $c_{x}$ and $c_{y}$ denote the damping coefficients, and $k_{x}$ and $k_{y}$ the stiffness coefficients along the *x*- and *y*-axes, respectively. Here, $\;m_{c}$ represents the equivalent mass of the Coriolis mass block, while the equivalent masses of the drive and sense modes are expressed as $m_{x}=m_{c}+m_{f_{x}}\;$ and $m_{y}=m_{c}+m_{f_{y}}$, respectively. $\;F_{d}sin(\omega_{d}t)$ corresponds to the driving force applied along the drive axis, $-2m_{c}\Omega_{z}$ represents the Coriolis force generated by the angular velocity about the *z*-axis during drive-mode operation, and $F_{y}$ is the feedback force in force-balance mode, used to counteract the sense-mode vibration along the *y*-axis.

An equivalent representation of Equation (1) is given by

(2) $$\begin{array}{l} \ddot{x}+\frac{\omega_{x}}{Q_{x}}\dot{x}+\omega_{x}^{2}x=\frac{F_{d}\sin(\omega_{d}t)}{m_{x}}\\ \ddot{y}+\frac{\omega_{y}}{Q_{y}}\dot{y}+\omega_{y}^{2}y=\frac{-2m_{c}\Omega_{z}}{m_{y}}A_{x}\omega_{d}\sin(\omega_{d}t)+F_{y}, \end{array}$$

where $\omega_{x}$ and $\omega_{y}$ denote the resonance frequencies of the drive and sense modes, $Q_{x}\;$ and $Q_{y}$ are the corresponding quality factors, and $A_{x}$ represents the amplitude of the drive-mode vibration.

### 2.2. Capacitive Feedthrough Model

In MEMS gyroscopes, electrostatic excitation is a widely adopted actuation method, often realized through a differential push-pull configuration. This approach involves applying two sinusoidal signals, possessing identical amplitude and opposing phase, to the drive electrodes. The resulting feedback force $F_{y}$ can be expressed as

(3) $$\begin{array}{ll} F_{y} & =\frac{1}{2}\frac{\partial C_{y}}{\partial y}[{\left(V_{bias}+V_{ac}\sin(\omega_{d}t)\right)}^{2}-{\left(V_{bias}-V_{ac}\sin(\omega_{d}t)\right)}^{2}]\\ & =2\frac{\partial C_{y}}{\partial y}V_{bias}V_{ac}\sin(\omega_{d}t), \end{array}$$

where $c_{y}\;$ denotes the capacitance of the drive combs in the sense mode, $V_{bias}$ refers to the DC voltage applied to the proof mass, and $V_{ac}$ signifies the amplitude of the AC excitation signal applied to the fixed electrodes.

**Figure 2:**
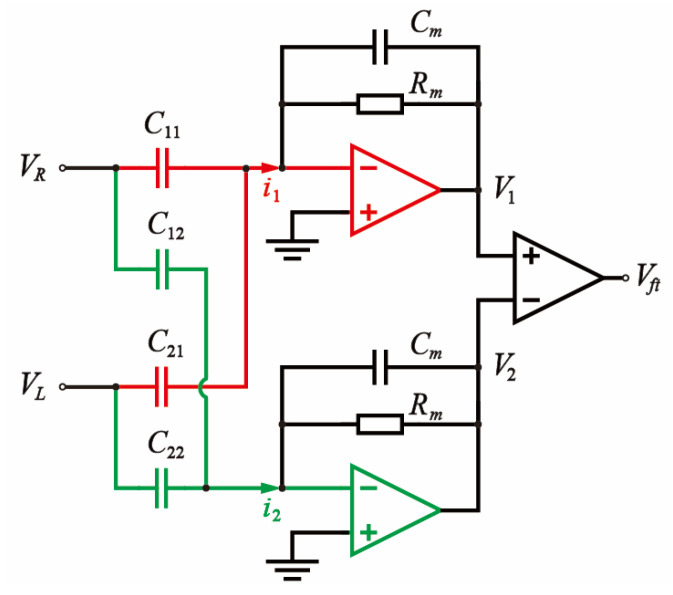
Feedthrough Capacitance Topology Model.

Under electrostatic actuation and capacitive sensing, the drive signal can couple into the sensing path via parasitic capacitance between the drive and sense electrodes, thereby introducing interference into the readout. The parasitic capacitance topology has been thoroughly investigated in previous studies [15,16,17]. As the dominant feedthrough originates from capacitive coupling between the drive and sense electrodes in the sense mode, the feedthrough capacitance model is accordingly simplified, as illustrated in Figure 2.

Figure 2 illustrates the labeling convention used for feedthrough capacitances: the first subscript indicates the polarity of the drive electrode, and the second denotes that of the sense electrode (“1” represents the positive AC signal applied to the electrode, and “2” represents the negative AC signal). The differential excitation signals are defined as $V_{R}=V_{dr}=V_{ac}\sin\left(\omega_{d}t\right),\;V_{L}=-V_{dr}$, where $V_{ac}\;$ denotes the amplitude of the excitation signal, and $\omega_{d}$ its angular frequency. These signals are applied to the gyroscope’s drive combs in an anti-phase configuration to realize push–pull actuation. The feedback resistor of the charge-sensitive amplifier (CSA) is denoted by $R_{m}$, and the resulting feedthrough voltage after differential amplification is represented by $V_{ft}$. The transfer function of the charge-sensitive amplifier (CSA) is given by

(4) $$G_{\mathrm{CSA}}(s)=-\frac{R_{m}}{1+sR_{m}C_{m}}.$$

The CSA feedback resistor and gain capacitor are designed to satisfy $1/R_{m}\ll \omega C_{m}$, where ω denotes the frequency of variation in the sensing capacitance [18]. Based on the equivalent circuit model [19], the corresponding relationship between the feedthrough and drive signals is derived as

(5) $$V_{ft}(s)=-V_{dr}(s)\frac{C_{11}-C_{21}-C_{12}+C_{22}}{C_{m}}.$$

Equation (5) indicates that, under conventional excitation schemes, the feedthrough signal possesses the same frequency and phase as the excitation signal. This spectral coincidence prevents effective separation from the gyroscopic vibration response in the frequency domain, thereby posing a fundamental challenge to feedthrough suppression.

## 3. Feedthrough Suppression Scheme Based on Mixed-Frequency Excitation

### 3.1. Theoretical Analysis

This section presents a feedthrough suppression scheme based on mixed-frequency excitation, along with its underlying modulation principle. In this approach, a modulated signal is applied to drive the harmonic oscillation of the gyroscope, which concurrently shifts the feedthrough component to a higher frequency band. As a result, the desired signal and feedthrough interference become spectrally separable, facilitating subsequent suppression. The excitation signal is defined as follows:

(6) $$\begin{array}{l} V_{R}=V_{ac}\sin(\omega_{d}t)\cdot q(t)+b(t)\\ V_{L}=V_{ac}\sin(\omega_{d}t)\cdot q(t)+2V_{bias}-b(t), \end{array}$$

where q(t) is a chopper signal applied to modulate the excitation path. It has a 50% duty cycle, an amplitude of ±1, and a period of Tₛ [20]. The bias injection signal b(t) shares the same periodicity. The definitions of q(t) and b(t) are given as follows:

(7) $$q(t)=\left\{\begin{array}{l} 1,\;\;\;\;\;0\leq t<0.5{Τ}_{s}\\ -1,\;\;\;0.5{Τ}_{s}\leq t<{Τ}_{s}, \end{array}\right.$$

(8) $$b(t)=\left\{\begin{array}{l} \delta V_{bias},\;0\leq t<0.5{Τ}_{s}\\ 0,\;\;\;\;\;\;\;\;\;\;0.5{Τ}_{s}\leq t<{Τ}_{s}. \end{array}\right.$$

Given that q^2^(t) = 1, the corresponding electrostatic force on the drive combs is expressed as

(9) $$\begin{array}{cc} F_{el}(t) & =\frac{1}{2}\frac{\partial C_{y}}{\partial y}[{\left(V_{ac}\sin(\omega_{d}t)q(t)+b(t)-V_{bias}\right)}^{2}-\;{\left(V_{ac}\sin(\omega_{d}t)q(t)-b(t)+V_{bias}\right)}^{2}]\\ & =2\frac{\partial C_{y}}{\partial y}V_{ac}V_{bias}\sin(\omega_{d}t)\left\{\begin{array}{l} (\delta -1),0\leq t<0.5{Τ}_{s}\\ 1,\;\;\;\;\;\;\;\;\;\;\;\;0.5{Τ}_{s}\leq t<{Τ}_{s}. \end{array}\right. \end{array}$$

For δ = 2, the electrostatic force produced by the mixed-frequency excitation signal is indistinguishable from that generated by the conventional excitation method. Under mixed-frequency excitation, both the closed-loop control input signals and the electrostatic forces from the drive electrodes remain the same as those in the conventional excitation scheme. As a result, the effective displacement response of the resonator remains unaltered, thereby confirming that the mixed-frequency excitation method theoretically maintains the linear characteristics inherent to the conventional excitation approach without necessitating additional demodulation circuitry [21].

According to the feedthrough capacitance model depicted in Figure 2, the feedthrough current $i_{1}(t)$ generated at the sense electrode under the mixed-frequency excitation scheme is given by

(10) $$i_{1}(t)=(C_{11}-C_{21})\frac{d}{dt}(V_{ac}\sin(\omega_{d}t)\cdot q(t))+(C_{11}+C_{21})\frac{d}{dt}b(t).$$

Expanding the square wave signals from Equation (10) into their Fourier series yields

(11) $$\begin{array}{l} i_{1}(t)=(C_{11}-C_{21})\frac{d}{dt}\{\frac{2V_{ac}}{\pi }\sum_{n=0}^{\infty }\frac{1}{2n+1}[\cos((\omega_{d}-(2n+1)\omega_{s})t)-\cos((\omega_{d}+(2n+1)\omega_{s})t)]\}+\\ \;\;\;\;\;\;\;\;\;(C_{11}+C_{21})\frac{d}{dt}\{V_{bias}+\frac{4V_{bias}}{\pi }\sum_{n=0}^{\infty }\frac{1}{2n+1}\sin((2n+1)\omega_{s}t)\}, \end{array}$$

where n = 1, 2, 3, …, ∞, and $\omega_{s}$ = 2π/$T_{s}$ denotes the angular frequency of the square wave. Equation (11) demonstrates that when a mixed-frequency excitation signal is employed, the resultant feedthrough current is modulated to the frequency bands of $\omega_{d}\pm (2n+1)\omega_{s}$ and n$\omega_{s}$. If $\omega_{s}$ is set to be significantly higher than the gyroscope’s operational frequency, the feedthrough signal and the desired signal can be effectively separated through filtering.

Equation (6) is expressed as follows:

(12) $$V_{R}=\left\{\begin{array}{l} V_{ac}\sin(\omega_{d}t)+2V_{bias}\;,\;\;\;\;\;\;0\leq t<0.5T_{s}\\ -V_{ac}\sin(\omega_{d}t)\;,\;\;\;\;\;\;\;\;\;\;\;\;\;\;0.5T_{s}\leq t<T_{s}, \end{array}\right.$$

(13) $$V_{L}=\left\{\begin{array}{l} V_{ac}\sin(\omega_{d}t)\;,\;\;\;\;\;\;\;\;\;\;\;\;\;\;\;\;\;\;\;\;0\leq t<0.5T_{s}\\ -V_{ac}\sin(\omega_{d}t)+2V_{bias}\;,\;\;\;\;\;\;\;\;0.5T_{s}\leq t<T_{s}. \end{array}\right.$$

**Figure 3:**
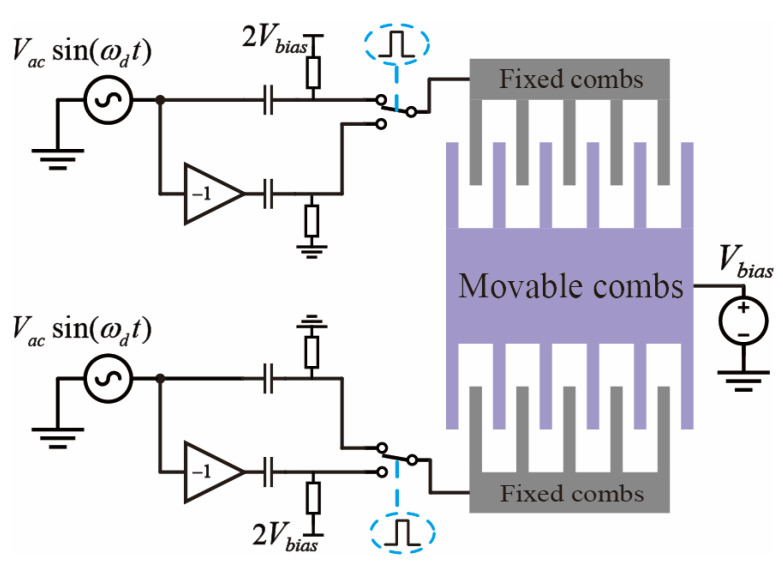
Mixed-frequency excitation signal generator.

In conformity with Equations (12) and (13), the mixed-frequency excitation signal generation circuit is configured as depicted in Figure 3. This circuit accepts a sinusoidal input signal at the gyroscope’s operational frequency [22]. The input signal is subsequently divided into two differential paths, thereby enabling a mixing operation facilitated by single-pole double-throw (SPDT) analog switches. Both switches maintain an identical initial position and are governed by a common square wave signal. Prior to the chopping operation at the switch nodes, one of the sinusoidal paths is subjected to a DC bias, precisely twice the magnitude of the center plate voltage, applied via a pull-up resistor. Concurrently, a coupling capacitor is employed to preclude the leakage of this DC bias into the preceding circuitry.

### 3.2. Mixed-Frequency Excitation Structure and Control System

**Figure 4:**
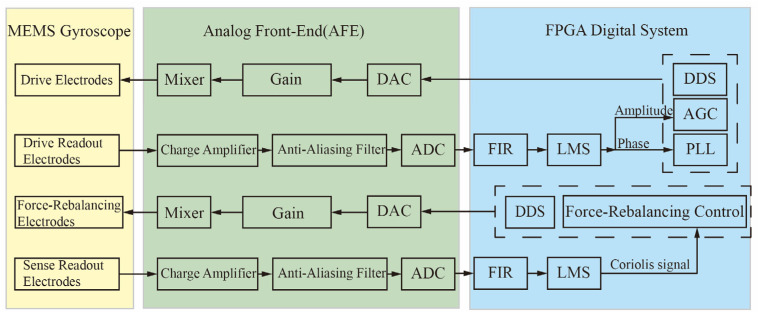
Mixed-Frequency Excitation Structure and Control System.

Figure 4 illustrates the Mixed-Frequency Excitation Structure and Control System constructed based on the mixed-frequency excitation signal generation circuit. A Phase-Locked Loop (PLL) and an Automatic Gain Control (AGC) scheme are employed to achieve closed-loop control of the gyroscope’s drive mode. Simultaneously, a force-rebalancing scheme is adopted to implement closed-loop sensing. A mixed-frequency module is integrated into the drive/force-rebalancing excitation signal generation circuit. In the readout path, an anti-aliasing low-pass filter is employed to preliminarily filter the mixed-frequency feedthrough signal, thereby ensuring its separation from the desired signal and precluding aliasing during ADC sampling. Upon its entry into the digital processing domain, the digitized signal is subjected to a Finite Impulse Response (FIR) bandpass filter for the further suppression of the feedthrough. Ultimately, a Least Mean Squares (LMS) demodulation algorithm is utilized to extract the control parameters for the drive and sense loops, with a Proportional-Integral (PI) controller executing the closed-loop control.

## 4. Analysis of Non-Idealities

The mixed-frequency signal generation circuit utilizes an op-amp based inverter to convert the sinusoidal signal into a differential signal. Stemming from non-idealities such as the operational amplifier’s gain-bandwidth product and the precision of the feedback resistor, the differential signal exhibits an amplitude mismatch of (1 − α) and a phase mismatch of β. This consequently degrades the quality of the mixed-frequency signal. The resulting non-ideal mixed-frequency excitation signal is thus defined as

(14) $$V_{R}=\left\{\begin{array}{l} \alpha V_{ac}\sin(\omega_{d}t+\beta )+2V_{bias}\;,\;0\leq t<0.5T_{s}\\ -V_{ac}\sin(\omega_{d}t)\;,\;\;\;\;\;\;\;\;\;\;\;\;\;\;\;0.5T_{s}\leq t<T_{s}, \end{array}\right.$$

(15) $$V_{L}=\left\{\begin{array}{l} \alpha V_{ac}\sin(\omega_{d}t+\beta )\;,\;\;\;\;\;\;\;\;\;\;\;\;\;0\leq t<0.5{Τ}_{s}\\ -V_{ac}\sin(\omega_{d}t)+2V_{bias}\;,\;\;\;\;\;\;\;\;0.5{Τ}_{s}\leq t<{Τ}_{s}. \end{array}\right.$$

First, we analyze the impact of these mismatches on the excitation performance. Based on the principle of electrostatic actuation, the electrostatic force generated by the excitation signal is given by

(16) $$F_{el}(t)=\frac{2V_{ac}V_{bias}\partial C_{y}}{\partial y}\left\{\begin{array}{l} \alpha \sin(\omega_{d}t+\beta ),0\leq t<0.5{Τ}_{s}\\ \sin(\omega_{d}t),\;\;\;\;\;\;\;0.5{Τ}_{s}\leq t<{Τ}_{s}. \end{array}\right.$$

Its Fourier series expansion is given by

(17) $$\begin{array}{l} F_{el}(t)=\frac{2V_{ac}V_{bias}\partial C_{y}}{\partial y}\{\frac{1}{2}\left(\left(\alpha \cos\beta +1\right)\sin(\omega_{d}t)+\alpha \cos(\omega_{d}t)\sin\beta \right)+\\ \left.\sum_{n=0}^{\infty }\frac{2}{\pi (2n+1)}\left[\begin{array}{l} \left(\frac{1}{2}\alpha \cos\beta -\frac{1}{2}\right)\left[\cos\left((2n+1)\omega_{s}t-\omega_{d}t\right)-\cos\left((2n+1)\omega_{s}t+\omega_{d}t\right)\right]\\ +\frac{1}{2}\alpha \sin\beta \left[\sin\left((2n+1)\omega_{s}t-\omega_{d}t\right)+\sin\left((2n+1)\omega_{s}t+\omega_{d}t\right)\right] \end{array}\right]\right\}. \end{array}$$

With a frequency counter, it was revealed that the differential signal’s phase mismatch (β) was approximately 2°, while the amplitude mismatch (1 − α) was about 1%. Under non-ideal conditions, the mixed-frequency excitation signal generates an electrostatic force that contains high-frequency components according to Equation (17). After normalization, the maximum amplitude was calculated to be −53.68 dB. Given that the gyroscope utilized in this study possesses a high quality factor of 200,000, its sensitivity to high-frequency components is minimal. As a result, the influence of these components can be reasonably neglected in subsequent analysis.

To further investigate the impact of non-ideal factors on the suppression of feedthrough interference, the non-ideal mixing excitation signal $V_{R}$ is analyzed via Fourier decomposition.

(18) $$\begin{array}{l} V_{R}=\sum_{n=0}^{\infty }\frac{\alpha V_{ac}\sin\beta }{\pi (2n+1)}\left[\sin\left((2n+1)\omega_{s}t-\omega_{d}t\right)+\sin\left((2n+1)\omega_{s}t+\omega_{d}t\right)\right]\\ +\sum_{n=0}^{\infty }\frac{\alpha V_{ac}\cos\beta +V_{ac}}{\pi (2n+1)}\left[\cos\left((2n+1)\omega_{s}t-\omega_{d}t\right)-\cos\left((2n+1)\omega_{s}t+\omega_{d}t\right)\right]\\ +\sum_{n=0}^{\infty }\frac{4V_{bias}}{\pi (2n+1)}\sin((2n+1)\omega_{s}t)+V_{bias}+\frac{V_{ac}\alpha \sin\beta }{2}\cos(\omega_{d}t)+\frac{V_{ac}(\alpha \cos\beta -1)}{2}\sin(\omega_{d}t). \end{array}$$

Equation (18) shows that mismatch parameters introduce fundamental frequency residual. Based on the given parameter values, the cosine and sine components of the residual signal are calculated to be 0.017 and −0.005, respectively. After being coupled to the gyroscope readout end through the feedthrough capacitor, this residual cannot be effectively separated by the filtering circuit, thereby undermining the suppression of feedthrough interference.

**Figure 5:**
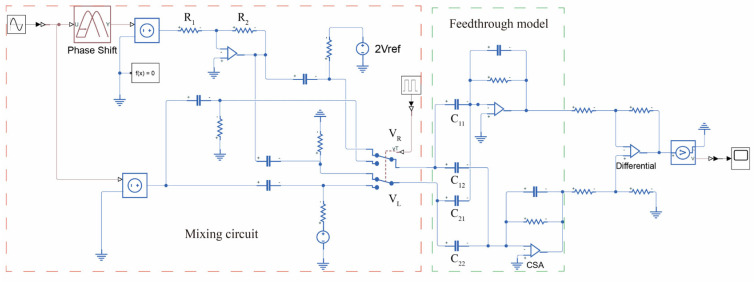
Non-ideal Mixed-frequency Excitation Circuit Simulation Model.

**Figure 6:**
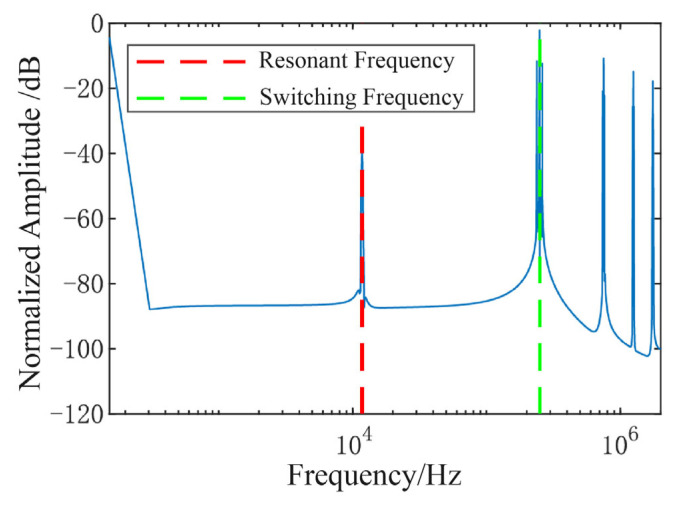
Spectrum of the non-ideal mixed-frequency excitation signal.

Figure 5 illustrates the simulation model of the mixed-frequency signal generation circuit under non-ideal conditions. Phase mismatch is implemented using a dedicated phase-shifting module, whereas amplitude mismatch is introduced by adjusting the resistance of R1 and R2. A sinusoidal signal with an amplitude of 1.5 V and a frequency matching the resonant frequency is then applied as the input. A Discrete Fourier Transform (DFT) analysis is performed on the mixed signal, with the resulting spectrum plot shown in Figure 6.

Simulation results reveal that, in the presence of non-idealities, the mixed-frequency excitation signal suffers from two dominant interferences: high-frequency artifacts introduced by modulation and fundamental residual at the resonant frequency.

**Figure 7:**
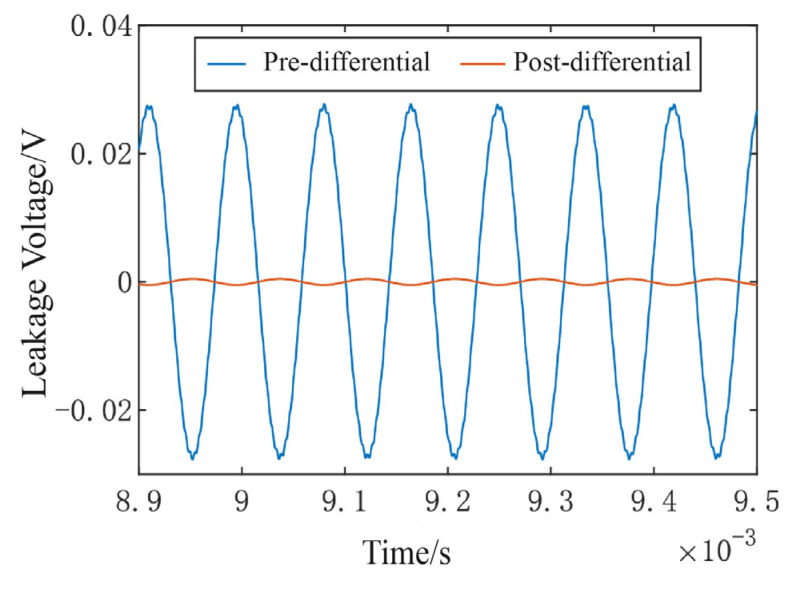
Pre- and post-differential fundamental frequency residual signal.

Under non-ideal conditions, the mismatch parameters of the excitation signals $V_{L}$ and $V_{R}$ are assumed to be identical, leading to fundamental frequency residual that exhibits a common-mode characteristic. Consequently, such a residual can be effectively suppressed using differential circuit techniques. To verify this behavior, a non-ideal mixed-frequency excitation signal is applied to the feedthrough capacitance model. After passing through a low-pass filter to eliminate high-frequency components, the residual fundamental frequency is extracted and analyzed. The corresponding simulation results are illustrated in Figure 7.

The results demonstrate that the differential circuit effectively suppresses fundamental frequency residuals caused by mismatches, reducing the amplitude of the feedthrough signal coupled into the readout path at the resonant frequency from 50 mV to less than 1 mV.

## 5. Experiments

**Figure 8:**
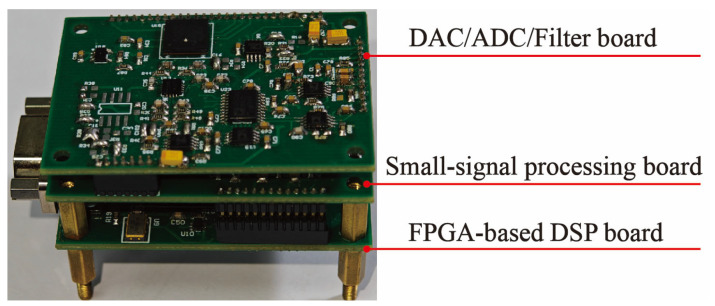
Photos of the MEMS gyroscope circuit.

To validate the feasibility of the mixed-frequency excitation approach, the measurement and control system outlined in Section 3 was employed to realize closed-loop control of both the drive and sense modes of the gyroscope. Figure 8 presents a photograph of the physical control circuit.

**Figure 9:**
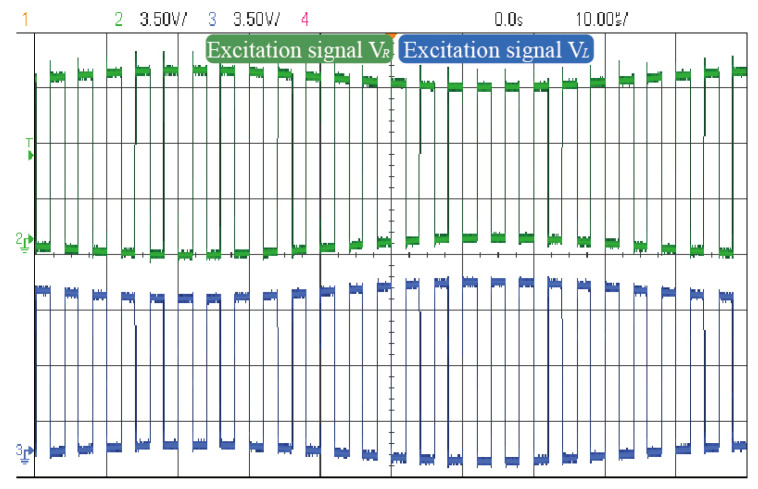
Mixed-frequency excitation signal waveform.

Figure 9 presents the oscilloscope-captured waveform of the mixed-frequency excitation signal. The mixed-frequency excitation signal exhibits an envelope modulation pattern, where the outer envelope shares the same frequency and amplitude as the input sinusoidal signal. The two mixed components are 180° out of phase in their envelope waveforms. Additionally, the square-wave carriers possess identical frequencies and maintain a 180° phase difference, in agreement with theoretical predictions.

**Figure 10:**
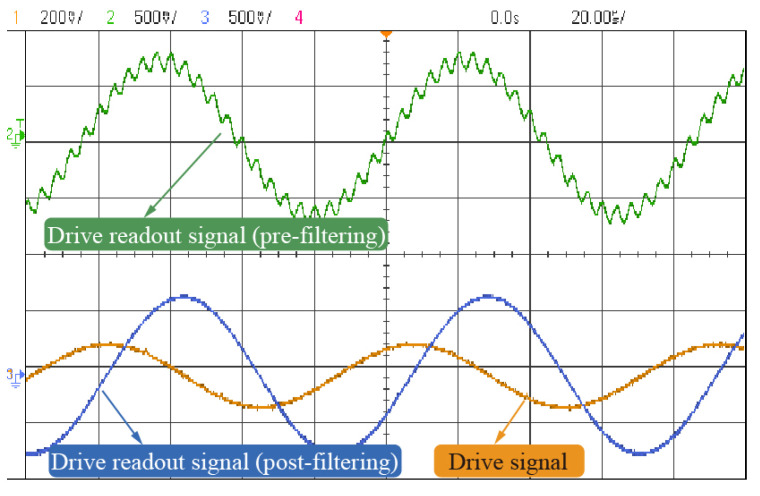
Readout Signal in Drive Circuit Pre- and Post-Filtering.

Figure 10 shows the waveform of the stabilized drive-mode signal before and after filtering. The comparison indicates that high-frequency feedthrough components are effectively suppressed. Additionally, the filtered readout signal exhibits a 90° phase shift with respect to the original drive signal, confirming that the gyroscope is operating at resonance.

**Figure 11:**
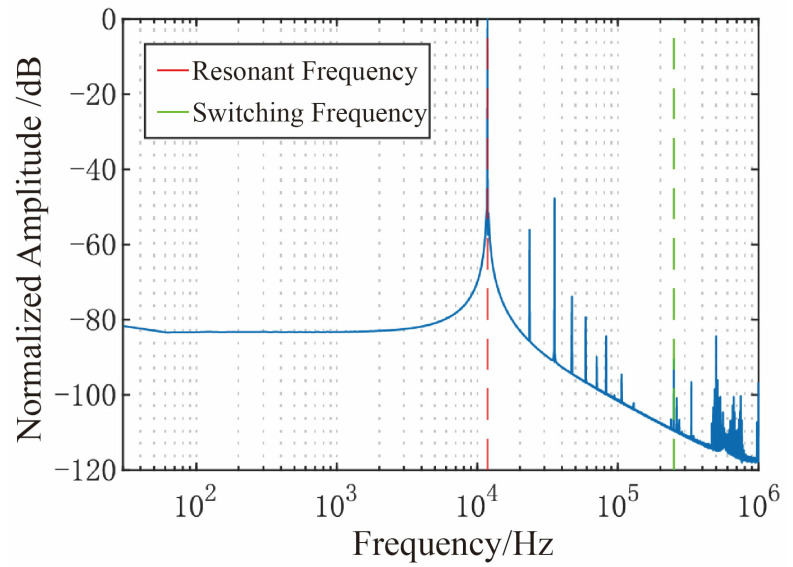
Drive Readout Signal Spectrum.

The filtered drive-mode readout signal was sampled and analyzed via Discrete Fourier Transform (DFT), and the resulting frequency spectrum is presented in Figure 11. The results indicate that high-frequency components are negligible, confirming the elimination of feedthrough interference and retention of the drive-mode signal.

**Figure 12:**
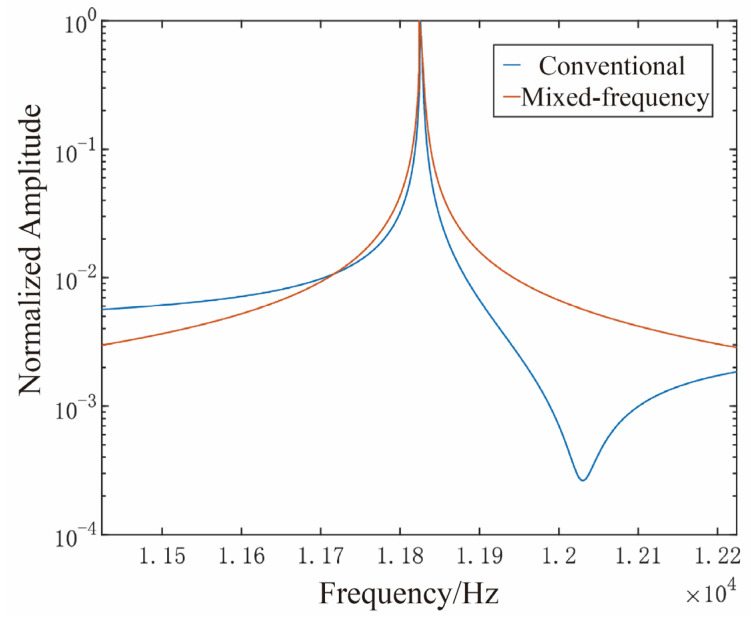
Open-loop frequency response curves for the two schemes.

Feedthrough interference adversely affects the resonant characteristics of MEMS gyroscopes, typically manifesting as an anti-resonant notch in the frequency response curve [23,24]. To validate the effectiveness of the proposed mixed-frequency excitation approach in suppressing feedthrough interference, open-loop frequency response measurements were carried out under both the conventional and mixed-frequency excitation conditions. During these experiments, the excitation amplitude was kept constant while the input frequency was linearly swept across a ±400 Hz range centered around the sense-mode resonant frequency. The corresponding frequency response curves are presented in Figure 12.

The results reveal that, under conventional excitation, the gyroscope exhibits a resonant peak accompanied by an anti-resonant zero near 12,023 Hz, indicative of significant feedthrough effects. In contrast, when subjected to the proposed mixed-frequency excitation, the anti-resonant feature disappears, and the frequency response presents a well-defined resonant peak. This observation confirms, from a frequency-domain perspective, the efficacy of the proposed approach in suppressing feedthrough interference.

In the gyroscope’s force-balanced mode, feedthrough interference compromises the scale factor [25,26] by causing the closed-loop scale factor to vary with the mode frequency split; the more pronounced the interference, the greater the resulting deviation.

**Figure 13:**
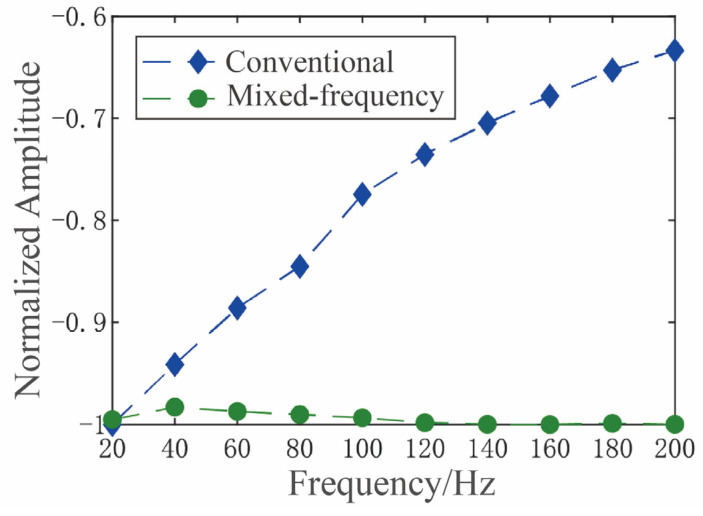
Normalized Scale Factor as a Function of Frequency Split for Two Schemes.

For the same MEMS gyroscope, both a conventional and a mixed-frequency excitation scheme were employed to implement force-balanced detection. The scale factor was then measured using a high-precision rate-position turntable under both control schemes, with the mode frequency split swept from 20 Hz to 200 Hz in 20 Hz increments. The resulting test data and the corresponding normalized scale factor variation curve are presented in Table 1 and Figure 13, respectively.

Under conventional excitation, the gyroscope’s scale factor decreased by 36.65% as the mode frequency split increased from 20 Hz to 200 Hz. In contrast, with the proposed mixed-frequency excitation, the maximum scale factor variation was limited to 1.7% over the same frequency split range—a 21-fold reduction in fluctuation. This demonstrates the effectiveness of the proposed approach in suppressing feedthrough interference from the perspective of scale factor stability.

The Zero-bias performance tests were conducted on MEMS gyroscopes under both a conventional and a mixed-frequency excitation approach. The tests yielded mean bias stability values of 0.2782 °/h and 0.2731 °/h for each approach, respectively, along with mean angular random walk values of 0.2757 °/√h/√Hz and 0.2405 °/√h/√Hz, respectively. The negligible difference in static performance between the two approaches indicates that the mixed-frequency excitation does not degrade the gyroscope’s static performance. The full test results are shown in Table 2.

## 6. Conclusions

This study presents a comprehensive theoretical investigation of feedthrough interference in MEMS vibratory gyroscopes. An equivalent relationship between the feedthrough signal and the excitation signal is derived, forming the theoretical foundation for a novel suppression strategy based on mixed-frequency excitation. By exploiting the quadratic dependence of electrostatic force on the excitation signal, the proposed method employs a switching circuit to modulate the excitation, effectively shifting the feedthrough interference to higher frequencies. This modulation enables frequency-domain separation between the interference and the desired vibratory signal.

The efficacy of the proposed approach was validated through a series of experimental evaluations. Open-loop frequency response measurements confirmed that mixed-frequency excitation effectively eliminates the anti-resonant artifacts introduced by feedthrough interference. Additionally, scale factor characterization revealed that the proposed method reduces the maximum fluctuation in the closed-loop scale factor by a factor of 21 compared to conventional excitation, confirming significant feedthrough suppression from both frequency and scale-factor perspectives. Finally, static performance testing demonstrated a mean bias stability of 0.3118 °/h and a mean angular random walk of 0.2443 °/√h/√Hz, underscoring the approach’s potential for high-performance MEMS gyroscope applications.

In perspective, the proposed strategy effectively mitigates the scale factor instability of MEMS gyroscopes under frequency tuning conditions, thereby improving measurement accuracy. This enhanced stability not only strengthens the reliability of MEMS gyroscopes in laboratory evaluations, but also provides significant benefits for practical applications such as high-precision navigation, inertial measurement systems, and autonomous platforms, where long-term accuracy and robustness against environmental variations are critical.

## Figures and Tables

**Table 1:** Scale factor variation with frequency split for both schemes.

Frequency Split(Hz)	Scale Factor (Conventional Excitation) (LSB/°/s)	Scale Factor (Mixed-Frequency) (LSB/°/s)
200	−12,005	−13,897
180	−11,301	−13,727
160	−10,636	−13,786
140	−10,147	−13,829
120	−9298	−13,873
100	−8829	−13,940
80	−8459	−13,962
60	−8140	−13,965
40	−7836	−13,949
20	−7605	−13,897

**Table 2:** Gyroscope static performance under both a conventional and a mixed-frequency excitation approach.

	Mixed-Frequency Excitation		Conventional	
	Bias Stability (°/h)	Angular Random Walk (°/h/√Hz)	Bias Stability (°/h)	Angular Random Walk (°/h/√Hz)
Test 1	0.3218	0.2319	0.2563	0.3121
Test 2	0.2349	0.2271	0.2471	0.2526
Test 3	0.2627	0.2626	0.3346	0.2625
Mean value	0.2731	0.2405	0.2782	0.2757

## Data Availability

The original contributions presented in this study are included in the article. Further inquiries can be directed to the corresponding author.
